# IMRT with ^18^FDG-PET\CT based simultaneous integrated boost for treatment of nodal positive cervical cancer

**DOI:** 10.1186/1748-717X-9-83

**Published:** 2014-03-25

**Authors:** Nikola Cihoric, Coya Tapia, Kamilla Krüger, Daniel M Aebersold, Bernd Klaeser, Kristina Lössl

**Affiliations:** 1 Department of Radiation Oncology, Bern University Hospital, and University of Bern, Freiburgstrasse, 3010 Bern, Switzerland; 2 Department of Nuclear Medicine, Bern University Hospital, and University of Bern, Bern, Switzerland; 3 University Bern, Institute for Pathology, Murtenstrasse 31, 3010 Bern, Switzerland

**Keywords:** Cervical cancer, Loco-regional lymph nodes, Intensity modulated radiotherapy, Simultaneous integrated boost

## Abstract

**Background:**

To evaluate toxicity and outcome of intensity modulated radiotherapy (IMRT) with simultaneous integrated boost (SIB) to the positive lymph nodes in patients with loco-regional advanced cervical cancer (LRACC).

**Methods:**

The study population comprised ten patients with ^18^FDG-PET\CT positive lymph nodes (LNs), who underwent chemoradiation with IMRT and SIB. A dose of 50.4 Gy, in daily fractions of 1.8 Gy, was delivered to primary tumor and draining LNs. Primary tumor received an additional external beam boost to a total dose of 55.8 Gy. A SIB of 62 Gy, in daily fractions of 2 Gy, was delivered to the ^18^FDG-PET\CT positive LNs. Finally, a high dose rate brachytherapy (HDRB) boost (15 – 18 Gy) was administered to the primary tumor. The primary goal of this study was to evaluate acute and early late toxicity and loco-regional control.

**Results:**

The median number of irradiated LNs per patient was 3 (range: 1–6) with a median middle nodal SIB-volume of 26.10 cm^3^ (range, 11.9-82.50 cm^3^). Median follow-up was 20 months (range, 12 to 30 months). Acute and late grade 3 toxicity was observed in 1 patient. Three of the patients developed a recurrence, one in the form of a local tumor relapse, one had a paraaortic LN metastasis outside the treated volume and the last one developed a distant metastasis.

**Conclusion:**

IMRT with SIB in the region of 18FDG-PET positive lymph nodes appears to be an effective therapy with acceptable toxicity and might be useful in the treatment of patients with locally advanced cervical cancer.

## Background

Despite advances in radiotherapy and combined treatment modalities, overall and disease free survival in LRACC remain unsatisfactory. A third of patients will develop recurrence within 2 years following therapy and 5-year relative survival for patients with affected regional LN is 57% [1-3]. In the absence of systemic metastasis, the most important predictive factor is the loco-regional LN status [4-6].

These facts lead to the hypothesis that effective treatment of loco-regional disease results in better disease control and longer survival. Additionally, effective loco-regional control may also prevent later complications caused by pelvic tumor growth. The primary tumor is treated with combination of a external beam radiotherapy (EBRT) and brachytherapy boost with doses that usually exceed 70 Gy (biological effective dose BED). Such dose levels are generally considered to be sufficient for local disease control and can be safely delivered due to the excellent conformity of brachytherapy. Due to the high level of toxicity, conventional EBRT technique (3-4-field-box) fails to deliver the necessary dose to treat gross disease in loco-regional LNs. A dose recommendation for treatment of clinically visible tumor manifestation in LNs is not well defined and varies between 55 and 60 Gy [7,8]. Higher conformity of IMRT helps to limit the dose to pelvic and abdominal organs at risk and results in a lower incidence of early and late toxicity [9]. Besides improving the therapeutic ratio, IMRT is capable of delivering different doses to different parts of the irradiated volume through dose painting or a simultaneous integrated boost (SIB) – a concept which has been studied in different tumor entities. Several authors have evaluated the use of SIB in the treatment of cervical cancer in preoperative settings and dose escalation in the parametric region [10-14].

Due to the many advantages of IMRT, we have developed a protocol addressing the treatment of ^18^FDG-PET\CT positive LNs using a SIB technique. The main goals of this study were to evaluate toxicity and effectiveness of the proposed therapy concept.

## Methods

### Patients

Patients with ^18^FDG-PET\CT positive pelvic or para-aortic LN were selected for treatment with radiotherapy delivered by SIB IMRT, according to our institutional standard protocol developed in 2009. Before therapy, all patients underwent a complete staging workup including medical history, physical and gynecologic examination, tumor biopsy, cystoscopy, manual rectal examination and anoscopy, magnetic resonance (MRI) and whole body ^18^FDG-PET\CT scan. Tumor staging was defined according to the International Federation of Obstetrics and Gynecology (FIGO) and TNM-UICC system. In the period between 03/2009 and 10/2010, ten patients were treated by IMRT SIB dose escalation to the region where ^18^FDG-PET\CT positive LNs were identified. No additional metastatic lymph nodes were detected on pelvic MRI. The median age at time of therapy was 53 years (range 42 to 83 years). Eight patients received concomitant weekly cisplatin chemotherapy (40 mg/m^2^). Two patients did not receive chemotherapy due to contraindications. Patient characteristics are summarized in Table 1. This study was approved by the local ethics committee (Kantonale Ethikkommission Bern).

**Table 1:** Patient characteristic

**Patient #**	**Age, years**	**FIGO stage**	**Histology (squamous cell carcinoma = SCC; adenocarcinoma = AC)**	**Tumor grade**	**Weekly concurrent cisplatine chemotherapy = +; without chemotherapy = -**
1	63	IVA	SCC	3	+
2	42	IIIB	SCC	2	+
3	42	IIIB	SCC	2	+
4	56	IIB	SCC	2	+
5	51	IIB	SCC	3	+
6	42	IIIB	SCC	2	-
7	71	IIIB	SCC	3	+
8	51	IIIB	AC	1	+
9	74	IIB	SCC	2	+
10	83	IIB	SCC	2	-

### Radiotherapy

A planning computed tomography (CT) scan was performed in supine position without contrast with slice thickness of 3 mm. Patients were instructed to come for the CT and radiotherapy with a full bladder. Image sets acquired by CT, diagnostic ^18^FDG-PET\CT and MRI were imported into the Eclipse Planning System (Varian Medical System, Paolo Alto, CA). We used “automatic matching algorithm”, with manual correction as needed. Registration quality was considered acceptable if dislocation of bony structures did not exceed 1 mm. The external beam radiotherapy was delivered using a dynamic multi-leaf linear accelerator with photon energies of 6 and 15 MV.

Two patients were treated with para-aortic RT. Eight patients were treated with a sequential IMRT boost in the primary tumor region with a median dose of 5.4 Gy (range 5.0 to 21.4 Gy). In one patient the external beam radiotherapy dose to the primary tumor was escalated to the total dose of 72 Gy because brachytherapy was not possible. Brachytherapy was not possible due to the obliteration of the cervical canal. The patient refused any surgical intervention including brachytherapy with needle insertion.

### Target volume delineation

#### *Tumor PTV*

The gross tumor volume of the cervix (GTVc) was defined as the visible macroscopic tumor based on all available clinical and imaging data. Clinical target volume for primary tumor area (CTVc) encompassed GTVc, uterus, parametria and upper third of vagina. In case of vaginal involvement CTVc expanded 2 cm into the vagina caudal of the tumor. The planning target volume of primary tumor (PTVc) was created using anisotropic expansion, considering cervical and surrounding structure movements. The PTVc was expanded to 15 mm in the antero-dorsal direction and 10 mm in the lateral direction. Asymmetrical margin for PTV was based on the fact that that the cervical cancer movements are not uniform in all directions, as showed in the work of Beadle et al. [15]. In the dorsal direction PTVc margin extended maximally to the posterior rectal wall and in frontal direction maximally 2 cm into the bladder.

#### *Nodal PTV and SIB volume*

The elective clinical target LN volume encompassed the vasa illiaca externa, interna and communis lymphatic chain to the aorta bifurcation and presacral LN area. In case of LN involvement at the level of a. communis or aortal LN, we extended the elective nodal volume to the level of renal arteries. A safety margin of 7 mm was added to construct the planning target volume (PTVn). PTVc and PTVn were merged to one single planning target volume (PTVsum).

Nodal gross tumor volume (GTVn) was based on the data acquired by ^18^FDG-PET\CT after assessments of other imaging modalities. Positive LNs were delineated separately as nodal gross tumor volume (GTVn). PTVsib was formed by adding a safety margin of 5 mm to the GTVn.

The prescription dose for PTVsum was 50.4 Gy delivered in 28 single fractions of 1.8 Gy. Upon completion of the first phase, three additional fractions were added to PTVc + PTVsib as external beam boost to a total dose of 55.8 Gy. Parallel to first and second phase PTVsib was irradiated with a dose escalation of 31 fractions of 2 Gy to a total dose of 62 Gy.

**Figure 1:**
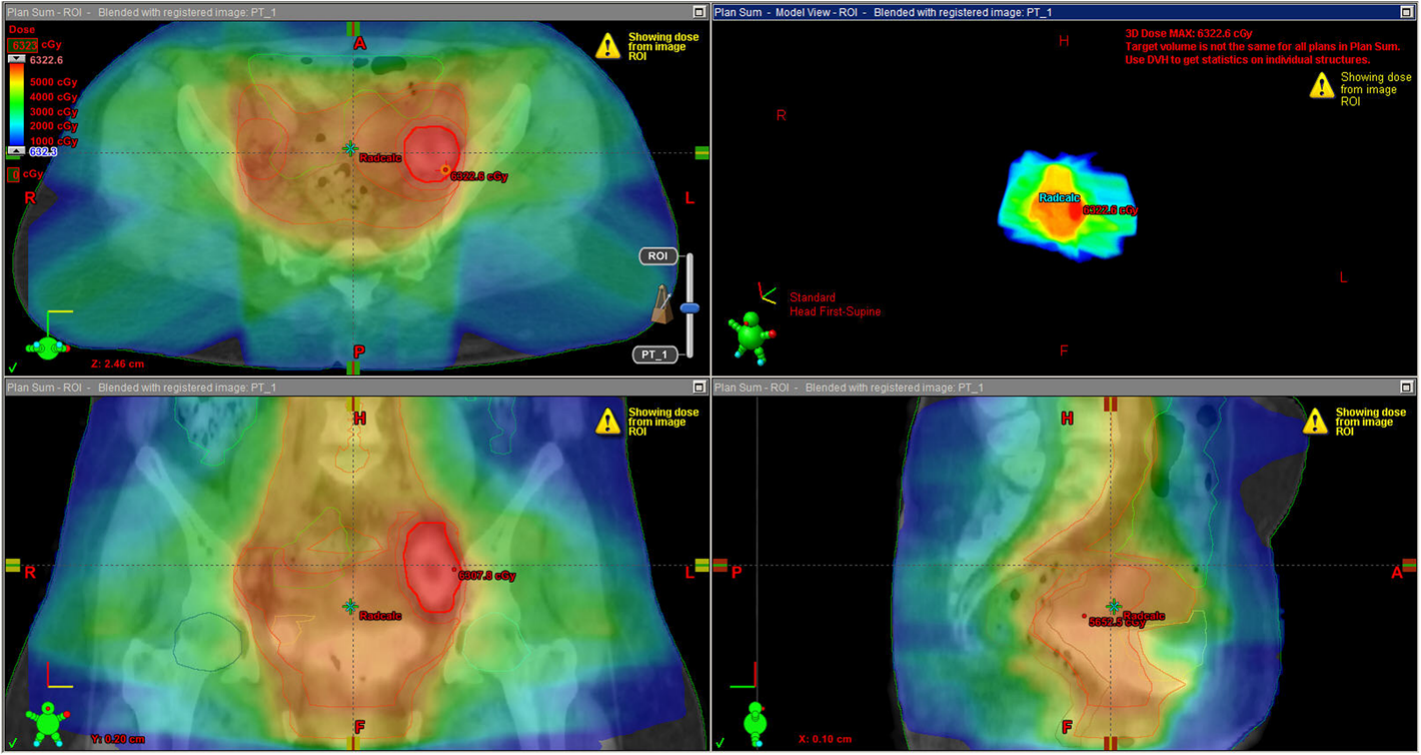
Example of a patient treatment plan with a metastatic iliac LN.

The example of treatment plan with a metastatic iliacal LN treated by SIB is shown on Figure 1.

### Constraints for organs at risk

Organs at risk were delineated on all axial slices. We delineated the rectum up to the sigmoid. The bowel was contoured to the level extending one vertebral body beyond the upper border of the PTV, including large and small intestines. The bladder and femoral heads were also contoured. Dose constraints for organs at risk were standardized as follows: 60% of rectal volume should receive no more than 50 Gy, 35% of bowel volume should receive no more than 35Gy, 50% of bladder volume should receive no more than 50 Gy and 10% of femoral heads volume should receive no more than 50 Gy.

### Brachytherapy

EBRT was followed by HDRB boost to the primary tumor, one week after completion of EBRT. Brachytherapy consisted of a total dose from 15 to 18 Gy delivered in 3 fractions with a single weekly fraction of 5 or 6 Gy, depending of the previous external beam total dose. We used a microSelectron® HDRB Unit and a Vienna Ring CT-MRI Applicator Set. Planning volume for HDRB was defined on the planning CT with applicators in treatment position. During planning we took into consideration data from an MRI scan performed during the last week of the EBRT treatment. Treatment volumes were delineated based on the Gynecological GEC-ESTRO Working Group recommendations [16].

### Toxicities

Acute and late toxicities were assessed according to the Common Terminology Criteria for Adverse Events Version 3.0 (CTCAE V3.0) scale. We defined acute toxicity as occurring during treatment or within the first 3 months after treatment end, whereas late toxicity was defined as any toxicity occurring later than 3 months after treatment. Acute toxicities were evaluated weekly during the treatment, at 6 weeks and 3 months after treatment completion. Late toxicities were evaluated 6 months after treatment and thereafter once a year. Evaluations of toxicities were done by a radiation oncologist.

### Follow-up

The initial tumor response was evaluated by a gynecologic oncologist 3 months after radiotherapy and every 3 months thereafter. We conducted a ^18^FDG-PET\CT 6 months after therapy for evaluation. Failure was defined as persistent disease or recurrence of disease following radiotherapy at any site. The date of failure was defined as the date of any sign of disease, either clinical or by imaging. The site of failure was recorded as local, nodal and distant. Furthermore, a distinction was established between in-field nodal failures or “out of field” nodal failures.

Patients without an event were censored at the date of last follow-up. Overall survival (OS) was calculated as time between the first day of radiotherapy to the date of death from any cause or last date of follow-up. Progression-free survival (PFS) was calculated as time between the first day of radiotherapy to the date of any sign of tumor relapse. Survival was analyzed using Kaplan-Meier plots. All analyses were carried out using SPSS V 20.0.

## Results

**Figure 2:**
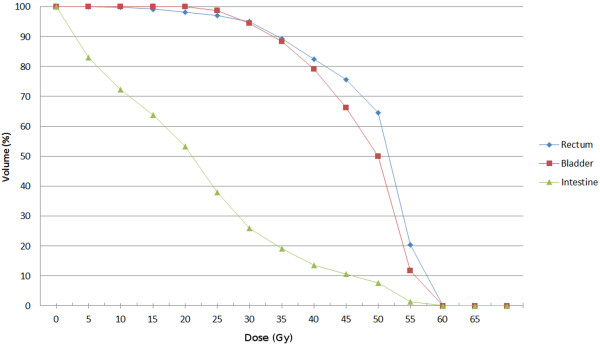
Dose Volume Histogram - median values for rectum, bladder and intestine volumes.

**Figure 3:**
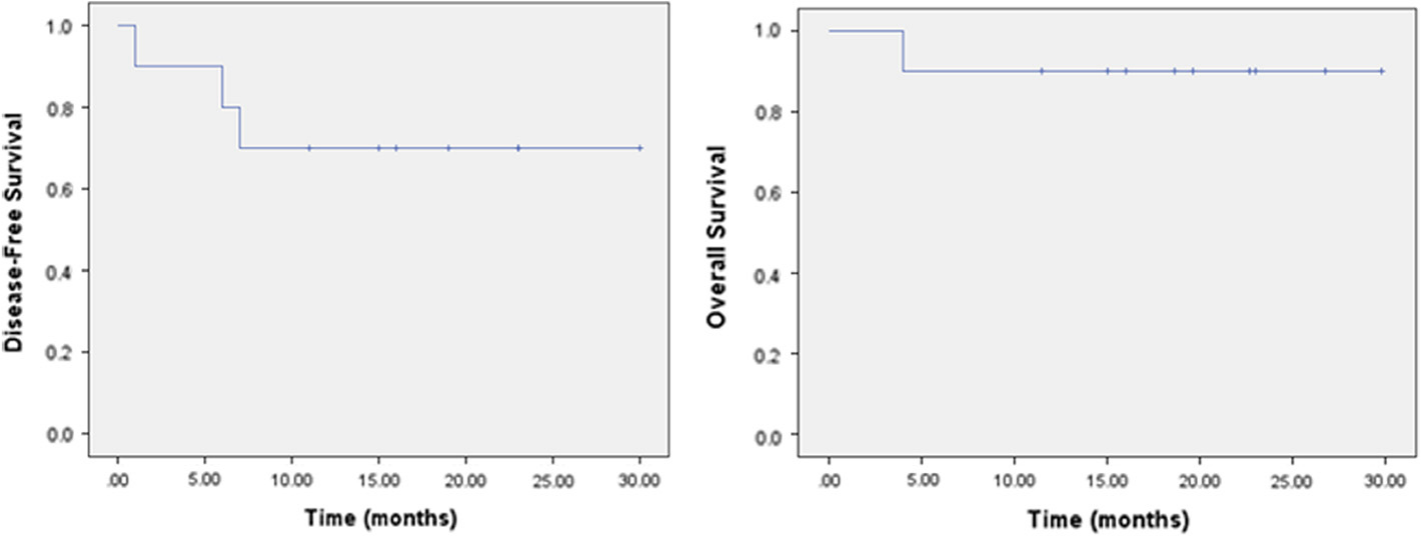
Kaplan-Meier curves representing disease free survival and overall survival.

A detailed overview of LN numbers, corresponding to SIB volumes and dose coverage for every patient is shown in Table 2. Dose volume histogram for rectum, bladder and intestine exposure (median of all 10 patients) is shown on Figure 2. Median follow-up time for all patients, excluding one who died three months after therapy, was 20 months (range 12 to 30 months). The overall-survival and disease-free survival curves are shown in Figure 3.

**Table 2:** Lymph nodes and corresponding radiotherapy volumes with dose coverage

**Patient #**	**Total number of **^**18**^**FDG PET-CT positive LNs**	**External and internal iliac LN**	**Common iliac LN**	**Para-aortic LN**	**SIB volumes (cm3)**	**Mean dose (Gy)**	**Min. dose (Gy)**	**Max. dose (Gy)**
**1**	3	1	0	2	71.30	60.03	53.19	63.06
**2**	1	1	0	0	29.50	62.14	59.60	63.20
**3**	2	2	0	0	14.60	61.16	58.10	61.81
**4**	3	3	0	0	17.20	61.97	59.49	62.80
**5**	3	2	1	0	22.70	60.96	58.79	61.81
**6**	6	1	2	3	82.50	63.67	54.20	66.87
**7**	3	2	0	1	18.40	61.40	58.66	62.74
**8**	2	2	0	0	33.60	61.22	59.20	62.31
**9**	1	1	0	0	11.90	60.92	57.48	62.92
**10**	2	2	0	0	33.50	61.20	57.84	62.89

Within the follow-up 7 patients remained diseases free. Two patients developed disease recurrence and one patient developed tumor persistence: One patient developed retroperitoneal LN metastases out of field 6 months after completion of RT. Twenty two months after completion of RT the same patient was diagnosed with local in-field recurrence in region of primary tumor (GTVc). One patient had tumor persistence diagnosed by ^18^FDG-PET\CT five months after RT. One patient developed systemic progression and para-aortic LN metastases out of field within one month after completion of radiotherapy.

### Treatment related toxicities

Eight patients had some form of unwanted therapy associated side effects resulting in minimal discomfort (grade 1 or 2 toxicity). One patient developed acute grade 3 toxicity in the form of cystitis which resulted in severe dysuria, polyuria and macroscopic hematuria. This patient was treated with conservative treatment and the symptoms resolved shortly after the therapy. An important confounding factor for this patient was an initially diagnosed urinary incontinence, rendering the patient incapable to maintain a full bladder during radiotherapy. The patient refused any form of catheterization. Therefore, the bladder filling could not be controlled during the radiotherapy. Dosimetric parameters for this patient were as follows: V30 = 99.7, V40 = 85.5, V50 = 14.0, V60 = 0.0; DMAX = 51.8 Gy. Overview of acute toxicities is presented in Table 3. One patient develop chronic vaginal dryness grade 3. We did not record any other late serious adverse event.

**Table 3:** Acute toxicity according to CTCAE V3.0

**Patient #**	**Upper gastrointestinal**	**Lower gastrointestinal**	**Urinary**	**Genital**	**Skin**
1	1	0	0	0	0
2	0	0	1	1	1
3	2	0	0	1	0
4	2	0	0	2	0
5	2	1	3	0	0
6	1	0	0	1	0
7	0	0	0	0	0
8	1	0	0	0	0
9	0	0	0	0	0
10	0	1	1	0	0
Gr 0	4	8	7	6	9
Gr 1	3	2	2	3	1
Gr 2	3	0	0	1	0
Gr 3	0	0	1	0	0

## Discussion

Presence of LN metastases in cervical cancer patients is a significant risk factor for disease recurrence. It has been demonstrated that that high proportion of patients with relapsed disease have a component of nodal failure. The reason for those unsatisfactory results could be attributed to insufficient dose delivered to the nodal region especially in the case of clinically suspected nodal metastasis, geographical miss or combination of both factors [7,17]. By reviewing currently available data, ^18^FDG-PET\CT shows more favorable results in the detection of regional disease when compared to the CT or MRI [4,18]. In addition, there is emerging evidence that the incorporation of modern molecular imaging (PET-CT) into the diagnosis and treatment contributes to better disease control [17]. This contribution can be reflected in better diagnosis of local and regional disease spread with consequent better delineation based on molecular data as shown in the work from Kidd et al. [17].

In an attempt to improve the therapeutic approach to nodal positive cervical cancer, we focused on a feasibility study of chemoradiation with IMRT and SIB to ^18^FDG-PET\CT positive LNs. One possible advantage in adopting IMRT relates to treatment planning with SIB based on data acquired from a 18FDG-PET\CT. With our treatment concept we have tried to avoid the aforementioned pitfalls with the incorporation of molecular data in the planning, and by delivering sufficient dose to the LN metastases. Moreover incorporation of SIB may shorten overall treatment time and can contribute to the disease control [19]. The data regarding this therapeutic approach is, however, limited. Marnitz S et al. described utilization of SIB delivered with tomotherapy in 40 patients with cervical cancer, focussing on use of the SIB for local cervical gross tumor. They treated the parametric region with SIB (single dose 2.12 Gy) to the 59.36 Gy. The region of interest was previously marked by surgical titan clips placement. The treatment results were satisfactory without excess in toxicity. Vandecasteele K. et al. (2009) report results of SIB implementation with intensity modulated arc therapy [10]. They created treatment plans for 4 patients with 18 FDG PET-CT positive lymph nodes. SIB volumes for nodes and primary treatment volume were delineated as one volume. The prescribed median dose to the GTV nodes was 60 Gy [10-14,20]. Compared to these published results, we applied sligthly higher total doses to 18 FDG PET-CT positive lymph nodes: We delivered 62 Gy, while remaining volume constraints for organs at risk.

The main concern when using dose escalation is the elevated number of acute serious adverse events. Several dosimetric studies have evaluated advantages of IMRT for cervical cancer in terms of dose reduction delivered to the organs at risk. Portelance et al. showed a 30 to 70% reduction in dose to the organs at risk with IMRT in comparison with conventional EBRT [21]. Roeske et al. achieved good target coverage with reduced intestinal dose [22]. Chan et al. and Kavanagh et al. demonstrated better protection of small bowel, rectum and bladder with IMRT over 4-Field and 3D conformal EBRT [23,24].

Current clinical experience to lymph nodes in cervical cancer is mainly based on dose regimes up to 50 Gy. Further dose escalation with conventional technique or even with 3D conformal therapy would put the bowel at risk. In case of SIB in pelvic and para-aortic regions, the risk of acute bowel injury could be an issue of concern. To date, there are generally no widely accepted dose constraints for organs and tissues and several proposals have been published by other authors. Gerszten K. et al. utilized a more aggressive approach in treatment of cervical cancer with extended field radiotherapy and added a 55 Gy boost to involved nodes. Authors proposed dose constraints as follows: Maximal dose for rectum, bladder and intestine should be ≤ 54 Gy. 40% of rectal volume and 50% of bladder volume should not receive more than 40Gy. Maximum of 35% intestinal volume should not receive more than 35 Gy [25]. Esthappan et al. treated PET positive para-aortic LNs with 60 Gy and elective nodal volume with 50 Gy. The DVH analysis showed that treatment plans irradiate approximately 50% of bowel with 25 Gy, less than 10% of bowel with 50 Gy and less than 1% received 60 Gy [26]. Although we have higher constraints for bladder and rectum we did not record higher incidence of toxicities compared with literature. Our dose constraints were easily achievable in most cases (Figure 2).

The 2 year disease free survival in our group (Figure 3.) is comparable with results from other authors. In the study from Hasselle et al. two year disease free survival (DFS) for patients with IIB-IVA cervical cancer treated with IMRT was reported as of nearly 70%. In the same study cumulative incidence of isolated pelvic failure (PF) and combined PF and distant failure was 8.6% and 10.1%, respectively [9]. Sushil B. et al. report a 51% 2 year DFS in patients treated with extended field IMRT in similar patients group [27]. In sequential paper Vandecasteele K. et al. (2012) report results of SIB utilization, with the same technique [10], in neoadjuvant settings in 30 patients [20]. Eleven patients had positive lymph nodes. Lymph nodes < 2 cm in diameter had 100% complete pathological response in contrast with lymph nodes ≥ 2 cm where complete response was achieved in 50% cases. In our cohort we achieved regional control of 100% within median follow up of 20 months. We do not conduct a surgical staging due to the fact that the morbidity rate following treatment is higher in patients receiving a combination of surgery and RT. Landoni et al. showed in a randomized trial that a subgroup of patients treated with postoperative RT had similar survival but a higher incidence of treatment related toxicities [28,29]. Data related to the laparoscopic staging and treatment of patients with cervical cancer is limited. Even though literature suggests different approaches this is still an open question. The available evidence for laparoscopic staging and treatment are mainly based on retrospective studies. One randomized trial showed no benefit of surgical vs. clinical approach in the staging and treatment of patients with cervical cancer [30]. The latest Cochrane Review found no evidence that pretreatment surgical para-aortic lymph node assessment for locally advanced cervical cancer is beneficial. However, they stated that the surgical approach could potentially have an adverse effect on survival [31]. A potential benefit of the laparoscopic staging is seen in low-stage cervix cancer (< FIGO IB2). The sensitivity of the laparoscopic staging is higher compared to staging with PET-CT [32]. In addition an NCI consensus recommends that a combination of surgery followed by radiotherapy should be avoided.

Late toxicities of the proposed treatment concept are an important issue. Gastrointestinal toxicities occur in ca. 10% of all patients and most occur within the first two years. The urological toxicities can rise up to 10% but their incidence can increase during time. New late toxicities can be detected up to 20 years after treatment. Our median follow-up of 20 months is limited in the detection of potential late toxicities [33].

Although being limited due to its small size and retrospective nature, the present study contributes to the notion that the application of a high dose of radiation in the region of ^18^FDG-PET\CT positive LNs by means of IMRT and SIB is feasible, with an acceptable profile of unwanted events and good loco-regional control, comparable with other published studies.

However, a prospective investigation with a larger sample size is needed to definitely confirm safety and efficiency of this therapeutic approach.

## Abbreviations

18FDG-PET\CT: Fluorodeoxyglucose positron emission tomography\computer tomography; CTCAE: Common terminology criteria for adverse events version 3.0; CTVc: Clinical target volume cervix; DVH: Dose volume histograms; EBRT: External beam radiotherapy; FIGO: Federation of obstetric and gynecology; GTVc: Gross tumor volume cervix; GTVn: Nodal gross tumor volume; HDR: High dose rate; IMRT: Intensity modulated radiotherapy; LN: Lymph node; LNs: Lymph nodes; LRACC: Loco-regional advanced cervical cancer; MRI: Magnetic resonance imaging; OS: Overall survival; PFS: Progression free survival; PTV: Planning target volume; PTVc: Planning target volume cervix; PTVsib: Planning target volume for simultaneous integrated boost; PTVsum: Total planning target volume; RT: Radiotherapy; SIB: Simultaneous integrated boost.

## Competing interests

The authors declare that they have no competing interests.

## Authors’ contributions

Each author had participated sufficiently in the work to take public responsibility for appropriate portions of the content. NC and KL design the study. The manuscript was written by NC, KL and DA. CT, KK, and BK collected the data and together with NC, KL and DA interpreted the data. All other authors helped and finally approved the manuscript.
